# Source Control in *Pseudomonas aeruginosa* Bloodstream Infections: An Overlooked Pillar in Reducing 30-Day All-Cause Mortality

**DOI:** 10.1093/ofid/ofag488

**Published:** 2026-09-24

**Authors:** Antonio Vena, Martina Bavastro, Marco Muccio, Michela Schenone, Silvia Corcione, Renato Pascale, Daniele Roberto Giacobbe, Simone Mornese Pinna, Bianca Pari, Francesca Giovannenze, Nicholas Geremia, Malgorzata Mikulska, Eleonora Taddei, Flavio Sangiorgi, Davide Fiore Bavaro, Vincenzo Scaglione, Silvia Sisto, Lucia Taramasso, Veronica Vassia, Michele Bartoletti, Francesco Giuseppe De Rosa, Maddalena Giannella, Pierluigi Viale, Matteo Bassetti, SITA GIOVANI (Young Investigators Group of the Società Italiana Terapia Antinfettiva, Accurso Giuseppe, Bavaro Davide Fiore, Chiappetta Stefania, Faliero Domenico, Fumarola Benedetta, Geremia Nicholas, Giovannenze Francesca, Maccaro Angelo, Marino Andrea, Merli Marco, Pagani Gabriele, Sardanelli Alessia, Scaglione Vincenzo, Tontodonati Monica, Vassia Veronica

**Affiliations:** Department of Health Sciences (DISSAL), University of Genoa, Genoa, Italy; Clinica Malattie Infettive, AOM - IRCCS Ospedale Policlinico San Martino, Genoa, Italy; Department of Health Sciences (DISSAL), University of Genoa, Genoa, Italy; Clinica Malattie Infettive, AOM - IRCCS Ospedale Policlinico San Martino, Genoa, Italy; Clinica Malattie Infettive, AOM - IRCCS Ospedale Policlinico San Martino, Genoa, Italy; Clinica Malattie Infettive, AOM - IRCCS Ospedale Policlinico San Martino, Genoa, Italy; Department of Medical Sciences, Infectious Diseases, University of Turin, Turin, Italy; Department of Infectious Diseases, Tufts University School of Medicine, Boston, Massachusetts, USA; Infectious Diseases Unit, IRCCS-Sant’ Orsola Polyclinic, Bologna, Italy; Department of Medical and Surgical Sciences, University of Bologna, Bologna, Italy; Department of Health Sciences (DISSAL), University of Genoa, Genoa, Italy; Clinica Malattie Infettive, AOM - IRCCS Ospedale Policlinico San Martino, Genoa, Italy; Department of Medical Sciences, Infectious Diseases, University of Turin, Turin, Italy; Department of Medical Sciences, Infectious Diseases, University of Turin, Turin, Italy; Department of Laboratory and Infectious Sciences, IRCCS A. Gemelli University Polyclinic Foundation, Rome, Italy; Unit of Infectious Diseases, Department of Clinical Medicine, Dell’Angelo Hospital, Venice, Italy; Department of Health Sciences (DISSAL), University of Genoa, Genoa, Italy; Clinica Malattie Infettive, AOM - IRCCS Ospedale Policlinico San Martino, Genoa, Italy; Department of Laboratory and Infectious Sciences, IRCCS A. Gemelli University Polyclinic Foundation, Rome, Italy; Department of Security and Bioethics-Infectious Diseases Section, Catholic University of the Sacred Heart, Rome, Italy; Department of Biomedical Sciences, Humanitas University, Milan, Italy; Infectious Disease Unit, IRCCS Humanitas Research Hospital, Rozzano, Milan, Italy; Infectious and Tropical Diseases Unit, Padua University Hospital, Padua, Italy; Department of Health Sciences (DISSAL), University of Genoa, Genoa, Italy; Clinica Malattie Infettive, AOM - IRCCS Ospedale Policlinico San Martino, Genoa, Italy; Clinica Malattie Infettive, AOM - IRCCS Ospedale Policlinico San Martino, Genoa, Italy; Infectious and Tropical Disease Unit, Mauriziano Umberto I Hospital, Turin, Italy; Infectious and Tropical Disease Unit, Civile Hospital, Ivrea, Italy; Department of Biomedical Sciences, Humanitas University, Milan, Italy; Infectious Disease Unit, IRCCS Humanitas Research Hospital, Rozzano, Milan, Italy; Department of Medical Sciences, Infectious Diseases, University of Turin, Turin, Italy; Infectious Diseases Unit, IRCCS-Sant’ Orsola Polyclinic, Bologna, Italy; Department of Medical and Surgical Sciences, University of Bologna, Bologna, Italy; Infectious Diseases Unit, IRCCS-Sant’ Orsola Polyclinic, Bologna, Italy; Department of Medical and Surgical Sciences, University of Bologna, Bologna, Italy; Department of Health Sciences (DISSAL), University of Genoa, Genoa, Italy; Clinica Malattie Infettive, AOM - IRCCS Ospedale Policlinico San Martino, Genoa, Italy

**Keywords:** bloodstream infections, mortality, *Pseudomonas aeruginosa*, source control

## Abstract

**Background:**

Appropriate antibiotic therapy is a key determinant of outcomes in *Pseudomonas aeruginosa* bloodstream infection (BSI), but the impact of adequate source control on prognosis has not been well defined in large cohorts.

**Methods:**

We conducted a retrospective multicenter study including hospitalized patients with *P. aeruginosa* BSI across 14 Italian hospitals between January 2021 and December 2022.

**Results:**

Overall, 639 BSI episodes were analyzed. The most frequent sources were an unknown source (31.8%), central venous catheter BSI (22.5%), hospital-acquired/ventilator-associated pneumonia (20.3%), urinary tract infections (11.4%), and intra-abdominal infections (6.9%). Source control was required in 34.0% of cases and was achieved early and adequately in 49.3% of these. The 30-day all-cause mortality rate was 23.9%. Mortality was significantly lower in patients who received early adequate source control (6.5%) compared with those without early adequate source control (23.6%) or in whom it was deemed unnecessary (28.4%) (*P* < .0001). In multivariable analysis, age, corticosteroid therapy, and septic shock were independently associated with increased mortality, whereas prior *P. aeruginosa* colonization, early adequate source control, and appropriate antibiotic therapy were protective. A propensity score–matched analysis confirmed the association between early adequate source control and reduced 30-day mortality (adjusted hazard ratio 0.15; *P* = .0024).

**Conclusions:**

Early adequate source control is a critical determinant of survival in *P. aeruginosa* BSI. Outcomes are optimized by a combined approach integrating timely source control with appropriate antibiotic therapy.

Adequate source control, along with appropriate antimicrobial therapy, is a key determinant of clinical outcomes in severe infections [1], including intravascular catheter-related [2], intra-abdominal [3], and urinary tract infections [4]. Multiple studies have underscored its prognostic value in bloodstream infections (BSIs) caused by a broad range of pathogens, such as Gram-positive (eg, *Staphylococcus aureus*, *Streptococcus* spp.) [5, 6] and Gram-negative bacteria (eg, *Enterobacterales*) [7], as well as fungi (eg, *Candida* spp.) [8].

*Pseudomonas aeruginosa* is a major nosocomial pathogen [9] and ranks as the third most common cause of hospital-acquired Gram-negative BSI [10], with mortality rates reaching up to 60% [11–16]. While the importance of timely and appropriate antimicrobial therapy has been well established [17, 18], the potential impact of early source control in *P. aeruginosa* BSI remains largely underexplored. To date, only a few small, single-center studies have specifically addressed this issue [19, 20], and none have systematically evaluated the interplay between appropriate antimicrobial therapy, adequate source control, and 30-day all-cause mortality.

This study aims to address this knowledge gap by identifying predictors of clinical outcomes in patients with *P. aeruginosa* BSI, with a particular focus on the impact of adequate source control on 30-day all-cause mortality.

## METHODS

This multicenter retrospective cohort study was conducted across 14 public hospitals in 8 Italian regions (Liguria, Piedmont, Lombardy, Veneto, Emilia-Romagna, Lazio, Apulia, and Sicily) over a 2-year period (2021–2022). The study was approved by the Ethics Committee of the City of Health and Science of Turin (Protocol No. 202/2023, PROT. No. 0066633).

The study population was identified using computerized microbiology laboratory databases from all participating centers. Inclusion criteria consisted of adult (≥ 18 years old) patients with at least one peripheral blood culture positive for *P. aeruginosa*. Exclusion criteria included polymicrobial BSI and recurrent *P. aeruginosa* BSI, with only the first episode per patient considered in the analysis.

### Data Collection

Baseline characteristics and process-of-care variables were retrieved from the automated hospital medical records, microbiology databases, and pharmacy databases of each participating hospital. Baseline characteristics included: age, gender, hospital ward at the time of *P. aeruginosa* BSI onset and underlying diseases, summarized using the Charlson Comorbidity Index [21]. Additional variables considered were immunosuppression (chemotherapy, corticosteroid therapy or other immunosuppressive therapy prior to BSI onset), neutropenia (absolute neutrophil count of <500/µL at the time of BSI onset), and invasive procedures performed within 30 days before *P. aeruginosa* BSI. These procedures included central venous catheter (CVC) placement, urinary catheterization, any type of surgery, and percutaneous endoscopic gastrostomy. Infection-related variables included previous *P. aeruginosa* colonization within 90 days, infection source, antimicrobial susceptibility profile of the *P. aeruginosa* isolate, severity of the disease at clinical presentation, antibiotic therapy, adequacy of source control, intensive care unit (ICU) admission after *P. aeruginosa* BSI onset, need for continuous renal replacement therapy (CRRT) after *P. aeruginosa* BSI onset, and 30-day all-cause mortality.

### Endpoint

The primary endpoint was 30-day all-cause mortality following the first positive blood culture for *P. aeruginosa.*

### Exposure Variable

The primary exposure variable was early adequate source control.

### Definition of Infection Source

The source of *P. aeruginosa* BSI was classified by the investigators at each participating center during data collection, before the statistical analysis, according to predefined study criteria and based on the clinical, microbiological, and imaging information available in the medical records. CVC-related BSI was defined by at least one of the following: (1) presence of exudate at the CVC exit site with isolation of *P. aeruginosa* (same phenotypic strain) from both the exudate and bloodstream; (2) a semi-quantitative catheter tip culture yielding >15 colony-forming units of the same *P. aeruginosa* strain isolated from blood, or (3) a differential time to positivity of ≥2 hours between blood cultures drawn from the CVC and a peripheral vein. The abdomen was considered to be the origin of the *P. aeruginosa* BSI when a patient had clinical and radiological evidence of abdominal infection and (1) a positive culture from an intra-abdominal site was obtained during surgery or needle aspiration or (2) no other apparent sources of *P. aeruginosa* BSI were detected. Urinary source was identified by *P. aeruginosa* isolation from urine culture or affected tissue, along with urologic conditions such as urinary tract manipulation or obstruction. Hospital-acquired pneumonia (HAP) or ventilator-associated pneumonia (VAP)–associated BSI was diagnosed when respiratory cultures yielded the same phenotypic strain, in the presence of clinical and radiological findings consistent with pneumonia [22]. BSI of unknown source was defined as a BSI for which no infectious source was identified by the participating investigators. This classification did not presume a specific pathogenesis and could therefore include neutropenic patients with possible gastrointestinal translocation, as well as patients with other unidentified sources.

### Early Adequate Source Control

The need for source control was classified retrospectively at each participating center according to the predefined criteria detailed below. All decisions regarding whether to perform source control had been made by the treating physicians during routine clinical care, independently of the study, based on the patient's clinical condition, procedural feasibility, and local practice. Source control was deemed necessary in clinical scenarios including the following: (1) removal of infected hardware (eg, endovascular prostheses, CVC) in BSIs associated with intravascular devices; (2) resolution of urinary tract obstruction in patients with urinary tract-related BSI; (3) surgical or image-guided drainage of infected collections and/or debridement of necrotic material in patients with intra-abdominal infections (eg, abscess, peritonitis), complicated pneumonia (eg, pneumonia with abscess or pleural empyema), and/or wound infections; (4) joint fluid drainage via arthrotomy or arthroscopy in cases of septic arthritis and/or osteomyelitis; and (5) cardiac surgery for endocarditis when indicated by current guidelines [23]. Source control was considered unnecessary in cases of BSI of unknown source or uncomplicated pneumonia. In all other cases, source control was deemed necessary. The timing of source control was defined as the interval between BSI onset and the performance of a definitive infection-resolving procedure. Early adequate source control was defined as intervention within 72 hours of *P. aeruginosa* BSI onset.

### Other Definitions

Bloodstream infection onset was defined as the date on which the first positive blood culture for *P.aeruginosa* was collected. Appropriate antibiotic therapy was defined as the administration of at least one antimicrobial agent at an adequate dosage with confirmed *in vitro* activity against the infecting isolate. The timing of appropriate antibiotic therapy was defined as the interval between *P. aeruginosa* BSI onset and the first intravenous dose of an active antimicrobial agent. Severity of illness was assessed by the local investigators at each participating center through review of the medical records, based on the presence of severe sepsis or septic shock at *P. aeruginosa* BSI onset [24]. The susceptibility profile of *P. aeruginosa* was classified as multidrug resistant (MDR), extensively drug resistant (XDR), or pandrug resistant (PDR), according to the standardized international definitions proposed by Magiorakos et al [25].

### Microbiological Studies

Blood cultures, organism identification, and susceptibility testing were performed at each participating center following standard operating procedures. Antimicrobial susceptibility was interpreted by local laboratories according to the European Committee on Antimicrobial Susceptibility Testing (EUCAST) recommendations [26].

### Statistical Analysis

Baseline demographic and clinical characteristics, as well as subsequent outcomes, were compared between patients who died within 30 days of *P. aeruginosa* BSI onset and those who survived. Categorical variables were expressed as counts and percentages, while continuous variables were reported as medians with interquartile ranges (IQRs). Comparisons were performed using the χ^2^ test or Fisher's exact test for categorical variables and the Wilcoxon rank sum test for continuous variables.

The impact of variables on 30-day all-cause mortality was evaluated through Cox proportional hazards regression, estimating both unadjusted hazard ratios (HRs) and multivariate-adjusted HRs (aHRs). Variables with *P* < .1 in unadjusted models were included in the multivariate analysis, along with characteristics associated with source control status (source control deemed not necessary, early adequate source control, inadequate source control), as identified through 3-group comparison. To identify independent predictors of 30-day all-cause mortality, a backward stepwise selection was then performed (model A). The same set of variables initially included in the backward stepwise selection for model A was re-entered into a new regression model, replacing fixed source control status with time-dependent source control to better address immortal time bias; a new backward selection was then performed to construct model B. In contrast, the set of variables retained in model A was used to build 3 additional models: model C, which applied a landmark analysis restricting inclusion to patients who were alive at day 4 after BSI onset (chosen because early adequate source control is defined within 72 hours), also to mitigate immortal time bias; model D, which included the study center as a shared frailty term to account for center-level heterogeneity; and model E, which evaluated source control (early adequate source control vs not), regardless of whether it was deemed necessary, as an additional robustness analysis. In addition, day-4 landmark Kaplan–Meier survival curves, including only patients who were alive at day 4 after *P.aeruginosa* onset, were generated to visually compare unadjusted 30-day survival between patients, stratified by source control status. Differences between the 3 groups were assessed using the log-rank test.

A sensitivity analysis restricted to patients with source control deemed necessary was conducted using the same methodology, to specifically address the subgroup for whom source control was indicated. Additionally, as part of the sensitivity analysis, the effect of early adequate source control on 30-day all-cause mortality was assessed through a propensity score (PS)–matching analysis. The PS was defined as the probability of receiving early adequate source control; it was estimated using logistic regression, including all variables with a *P* value of <.15 in the unadjusted comparison between patients with and without early adequate source control, as well as septic shock at BSI onset. Patients were matched 1:1 using nearest neighbor matching without replacement and a caliper of 0.1. Covariate balance was assessed using standardized differences. In an exploratory analysis, mortality was also compared between patients who received appropriate antibiotic therapy and those who did not, stratified by early source control adequacy, in the subgroup with source control deemed necessary. To reduce immortal time bias, only patients who were alive at day 4 after BSI onset were included.

Time to adequate source control, ICU admission, need for CRRT after *P. aeruginosa* BSI onset, and time to appropriate antibiotic therapy were treated as time-dependent covariates in the Cox regression models. The proportional hazards assumption was verified for all fixed-effect Cox regression models, using cumulative sums of martingale residuals and Kolmogorov-type supremum tests. Missing data among baseline variables were limited to 1 missing age value, which was imputed using the cohort median. A *P* value of ≤.05 was considered statistically significant, and all tests were 2-sided. Statistical analyses were conducted using SPSS version 28.0.1.0 (SPSS Software, Chicago, IL) and SAS software version 9.4 (SAS Institute Inc., Cary, NC).

## RESULTS

During the study period, 639 episodes of *P. aeruginosa* BSI were identified. Bloodstream infection of unknown source was the most frequent category, accounting for 203 episodes (31.8%), followed by CVC-related BSI (144 cases, 22.5%), HAP/VAP-related BSI (130 cases, 20.3%), urinary tract infection (73 cases, 11.4%), and intra-abdominal infection (44 cases, 6.9%). Among patients with BSI of unknown source, 36 (17.7%) were neutropenic. Their clinical characteristics are detailed in Supplementary Table 1.

Overall, source control was deemed necessary in 217 patients (34.0%) and was performed adequately and early in 107 of these cases (49.3%). The median time from *P. aeruginosa* BSI onset to adequate source control was 3 days (IQR 1–5). The baseline characteristics of the 3 patient groups are reported in Supplementary Table 2.

**Figure 1.**
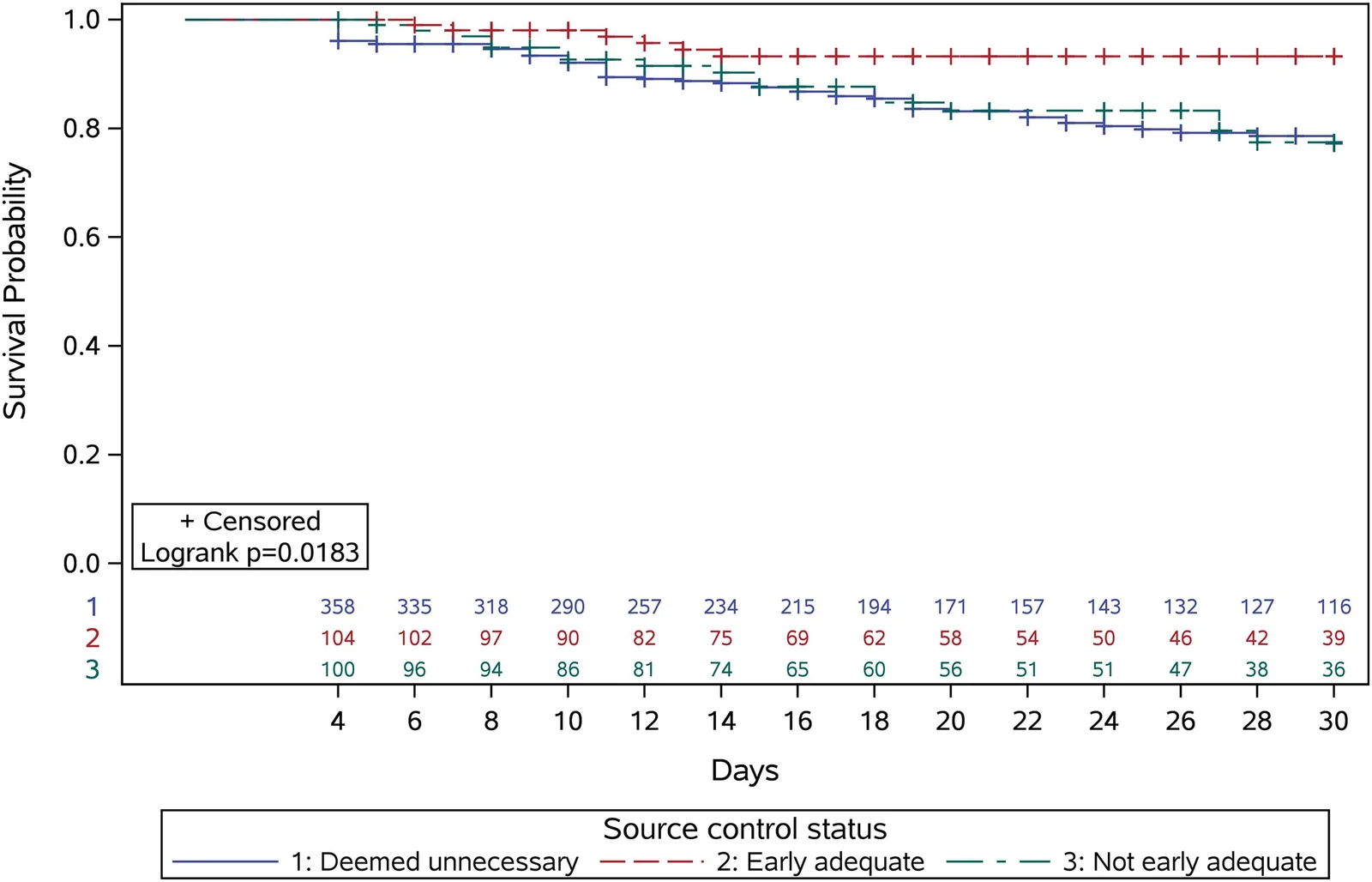
Landmark Kaplan–Meier curves including only patients who were alive at day 4 after bloodstream infection (BSI) onset, stratified by source control status (deemed unnecessary, early adequate, not early adequate).

Table 1 provides an overview of the entire cohort, including a comparison between patients who died and those who survived. Among the 639 patients, 153 (23.9%) died within 30 days. The unadjusted 30-day all-cause mortality rate was significantly lower in patients who received early adequate source control (6.5%), while no significant difference was observed between those who did not undergo early source control (23.6%) and those for whom source control was deemed not necessary (28.4%) (Supplementary Table 2). Univariate and multivariate analyses of factors associated with 30-day all-cause mortality are presented in Table 2. The backward stepwise multivariable analysis (model A) identified age (aHR 1.03; CI 1.02–1.04, *P* < .0001), corticosteroid therapy (aHR 1.99; CI 1.41–2.81, *P* = .0001) and septic shock at *P. aeruginosa* BSI onset (aHR 3.85; CI 2.77–5.35, *P* < .0001) as factors independently associated with higher mortality. Conversely, previous history of *P. aeruginosa* colonization (aHR 0.60; CI .40–.90, *P* = .0142), early adequate source control (aHR 0.26; CI .11–.60, *P* = .0016), and appropriate antibiotic therapy (aHR 0.27; CI .18–.40, *P* < .0001) were factors found to be protective against mortality. To account for potential immortal time bias affecting source control, models B and C were also performed. In particular, landmark analysis (model C) confirmed age (aHR 1.03; CI 1.01–1.05, *P* = .0005), septic shock (aHR 2.44; CI 1.51–3.96, *P* = .0003), prior *P. aeruginosa* colonization (aHR 0.52; CI .29–.91, *P* = .0227), early adequate source control (aHR 0.35; CI .14–.90, *P* = .0293), and appropriate antibiotic therapy (aHR 0.41; CI .21–.78, *P* = .0067) as significant predictors of 30-day all-cause mortality (Table 3; Figure 1). Including center as shared frailty did not alter the results of the model. Similarly, model E evaluating early adequate source control regardless of whether it was deemed necessary, confirmed the robustness of the main findings, as reported in Table 3.

**Table 1.** Demographic and Clinical Characteristics of the Entire Population With *Pseudomonas aeruginosa* BSI (N = 639), with a Comparison Between Alive and Dead Patients for All-Cause Mortality at 30 day

Variables	Total n = 639	Alive n = 486	Dead n = 153	*P* Value
Age (y), median (IQR)	68 (57–78)	66 (55–76)	72 (65–79)	**<**.**0001**
Male sex, n (%)	431 (67.5)	334 (68.7)	97 (63.4)	.2202
Hospital ward at the time of *P. aeruginosa* BSI onset, n (%)				.**0086**
Internal medicine	279 (43.7)	219 (45.1)	60 (39.2)	
Surgery	100 (15.7)	82 (16.9)	18 (11.8)	
Oncology-hematology	71 (11.1)	56 (11.5)	15 (9.8)	
ICU	183 (28.6)	127 (26.1)	56 (36.6)	
Others	6 (0.9)	2 (0.4)	4 (2.6)	
Underlying diseases, n (%)				
Cardiovascular disease	207 (32.4)	147 (30.3)	60 (39.2)	.**0387**
Neurological disease	179 (28.0)	134 (27.6)	45 (29.4)	.6585
Diabetes mellitus	125 (19.6)	93 (19.1)	32 (20.9)	.6285
Solid malignancy	133 (20.8)	95 (20.0)	38 (24.8)	.1599
Gastrointestinal disease	110 (17.2)	80 (16.5)	30 (19.6)	.3685
Hematological malignancy	104 (16.3)	82 (16.9)	22 (14.4)	.4662
Chronic renal failure	99 (15.5)	75 (15.4)	24 (15.7)	.9396
Chronic obstructive pulmonary disease	74 (11.6)	53 (10.9)	21 (13.7)	.3418
Hematopoietic stem cell transplant	35 (5.5)	29 (6.0)	6 (3.9)	.3322
Solid organ transplant	28 (4.4)	23 (4.7)	5 (3.3)	.4402
Liver cirrhosis	21 (3.3)	17 (3.5)	4 (2.6)	.5929
Splenectomy	10 (1.6)	8 (1.7)	2 (1.3)	1.0000**^g^**
HIV	8 (1.3)	7 (1.4)	1 (0.7)	.6873**^g^**
Charlson comorbidity index, median (IQR)	4 (2–6)	3 (2–5)	4 (2–6)	.**0082**
Corticosteroid therapy, n (%)	142 (22.2)	93 (19.1)	49 (32.0)	.**0008**
Chemotherapy, n (%)	82 (12.8)	60 (12.4)	22 (14.4)	.5119
Other immunosuppressive therapy, n (%)	72 (11.3)	59 (12.1)	13 (8.5)	.2139
Neutropenia, n (%)	70 (11.0)	51 (10.5)	19 (12.4)	.5062
Intermittent hemodialysis, n (%)	36 (5.6)	30 (6.2)	6 (3.9)	.2922
Invasive procedures^d^, n (%)				
Central venous catheter	411 (64.3)	319 (65.6)	92 (60.1)	.2149
Urinary catheter	419 (65.6)	311 (64.0)	108 (70.6)	.1342
Any surgery	172 (26.9)	133 (27.4)	39 (25.5)	.6482
Percutaneous endoscopic gastrostomy	40 (6.3)	32 (6.6)	8 (5.2)	.546
Previous *P. aeruginosa* colonization^e^, n (%)	171 (26.8)	141 (29.0)	30 (19.6)	.**0219**
Source of bloodstream infection^b^, n (%)				**<**.**0001**^g^
Unknown source	203 (31.8)	138 (28.4)	65 (42.5)	
CVC-related BSI	144 (22.5)	124 (25.5)	20 (13.1)	
HAP**/**VAP	130 (20.3)	89 (18.3)	41 (26.8)	
Urinary tract infections	73 (11.4)	67 (13.8)	6 (3.9)	
Intra-abdominal infections	44 (6.9)	31 (6.4)	13 (8.5)	
Skin and soft tissue infection	26 (4.1)	23 (4.7)	3 (2.0)	
Osteomyelitis	3 (0.5)	3 (0.6)	0 (0.0)	
Central nervous system infection	2 (0.3)	1 (0.2)	1 (0.7)	
Others^f^	14 (2.2)	10 (2.1)	4 (2.6)	
Susceptibility profile^c^, n (%)				.9074
MDR	57 (9.1)	42 (8.7)	15 (10.2)	
XDR	40 (6.4)	30 (6.2)	10 (6.8)	
PDR	7 (1.1)	6 (1.2)	1 (0.7)	
Clinical presentation, n (%)				**<**.**0001**
Sepsis	312 (48.8)	245 (50.4)	67 (43.8)	
Septic shock	129 (20.2)	65 (13.4)	64 (41.8)	
No sepsis	198 (31.0)	176 (36.2)	22 (14.4)	
Appropriate antibiotic therapy, n (%)	578 (90.5)	459 (94.4)	119 (77.8)	**<**.**0001**
Source control status, n (%)				**<**.**0001**
Deemed unnecessary	422 (66.0)	302 (62.1)	120 (78.4)	
Deemed necessary but not performed early	110 (17.2)	84 (17.3)	26 (17.0)	
Deemed necessary and adequately performed early	107 (16.7)	100 (20.6)	7 (4.6)	
Time to source control (d)^a^, median (IQR)	3 (1–5)	3 (1–5)	2.5 (1–4)	.4906
Outcomes, n (%)				
Need for ICU admission	23 (3.6)	14 (2.9)	9 (5.9)	.0822
Need for CRRT after *P. aeruginosa* BSI	20 (3.1)	9 (1.9)	11 (7.2)	.**0023**

The reported *P* values are derived from the Wilcoxon rank sum test for continuous variables and χ^2^ or Fisher's exact test for categorical variables. Bold values indicate statistical significance at the predefined level (α = .05). One patient alive at 30 d had missing age data, which was imputed using the median for subsequent analyses. The median, first and third quartiles, and *P* value reported in the table were identical before and after imputation. No other missing values were present in this table.

Abbreviations: BSI, bloodstream infection; CRRT, continuous renal replacement therapy; CVC, central venous catheter; ICU, intensive care unit; IQR, interquartile range; HAP, hospital-acquired pneumonia; HIV, human immunodeficiency virus; MDR, multidrug resistant; PDR, pandrug-resistant; VAP, ventilator-associated pneumonia; XDR, extensively drug resistant.

^a^Analysis conducted on a total of 172 patients who underwent source control during the study period.

^b^Test calculated using Monte Carlo simulation.

^c^Analysis conducted on a total of 629 patients with a known susceptibility profile.

^d^Within 30 d before *P. aeruginosa* BSI onset.

^e^Within 90 d before *P. aeruginosa* BSI onset.

^f^Includes mediastinitis, endocarditis, infection of spinal fixation hardware, community-acquired pneumonia, infection of prosthetic femur, lung abscess, pleural empyema, left ventricular assist device (LVAD) infection.

^g^Fisher's exact test.

**Table 2.** Univariable, Multivariable, and Backward Selection Multivariable Analyses of Factors Associated With 30-d All-Cause Mortality in the Entire Study Population (n = 639)

	Univariable		Multivariable		Multivariable Backward (Model A)	
Variables	HR (95% CI)	*P* Value	aHR (95% CI)	*P* Value	aHR (95% CI)	*P* Value
Age (y)	1.03 (1.02–1.05)	**<**.**0001**	1.03 (1.01–1.04)	**<**.**0001**	1.03 (1.02–1.04)	**<**.**0001**
Male sex	0.80 (.57–1.11)	.1792	0.84 (.60–1.18)	.3192	…	**…**
Cardiovascular disease	1.40 (1.01–1.94)	.**0424**	1.20 (.84–1.70)	.3155	…	…
Neurological disease	1.07 (.76–1.52)	.6899	…	…	…	…
Diabetes mellitus	1.07 (.73–1.58)	.7296	…	…	…	…
Solid malignancy	1.36 (.94–1.96)	.1021	…	…	…	…
Gastrointestinal disease	1.26 (.85–1.89)	.2494	…	…	…	…
Hematological malignancy	0.91 (.58–1.42)	.6705	…	…	…	…
Chronic renal failure	1.10 (.71–1.71)	.6585	…	…	…	…
Chronic obstructive pulmonary disease	1.31 (.83–2.08)	.2519	…	…	…	…
Hematopoietic stem cell transplant	0.67 (.29–1.50)	.3266	…	…	…	…
Solid organ transplant	0.72 (.30–1.75)	.4682	…	…	…	…
Liver cirrhosis	0.87 (.32–2.34)	.7749	…	…	…	…
Splenectomy	0.80 (.20–3.24)	.7585	…	…	…	…
HIV**^a^**						
Charlson comorbidity index	1.08 (1.02–1.13)	.**0044**	1.00 (.95–1.06)	.9862	…	**…**
Corticosteroid therapy	1.83 (1.30–2.57)	.**0005**	2.00 (1.41–2.86)	.**0001**	1.99 (1.41–2.81)	.**0001**
Chemotherapy	1.23 (.78–1.93)	.3710	…	…	…	…
Other immunosuppressive therapy	0.74 (.42–1.31)	.3031	…	…	…	…
Neutropenia	1.22 (.76–1.97)	.4165	…	…	…	…
Intermittent dialysis treatment	0.64 (.28–1.44)	.2797	0.51 (.22–1.17)	.1128	…	…
Central venous catheter^e^	0.73 (.53–1.02)	.0622	0.90 (.63–1.29)	.5686	…	…
Urinary catheter^e^	1.19 (.84–1.69)	.3253	…	…	…	…
Any surgery^e^	0.87 (.61–1.26)	.4674	…	…	…	…
Percutaneous endoscopic gastrostomy^e^	0.70 (.34–1.43)	.3284	…	…	…	…
Previous *P. aeruginosa* colonization^f^	0.57 (.38–0.85)	.**0059**	0.63 (.41–.96)	.**0320**	0.60 (.40–.90)	.**0142**
Septic shock	3.53 (2.56–4.87)	**<**.**0001**	4.18 (2.99–5.84)	**<**.**0001**	3.85 (2.77–5.35)	**<**.**0001**
Appropriate antibiotic therapy**^c^**	0.26 (.18–.38)	**<**.**0001**	0.29 (.20–.43)	**<**.**0001**	0.27 (.18–.40)	**<**.**0001**
Time to appropriate antibiotic therapy (d)**^d^**	0.89 (.76–1.03)	.1143	…	**…**	…	**…**
Source control status (vs deemed necessary but not performed early)	…	.**0001**	…	**<**.**0001**	…	**<**.**0001**
Deemed necessary and adequately performed early	0.26 (.11–.61)	.**0017**	0.24 (.10–.55)	.**0009**	0.26 (.11–.60)	.**0016**
Deemed unnecessary	1.33 (.87–2.03)	.1864	1.46 (.95–2.25)	.0876	1.49 (.97–2.29)	.0656
Time to source control (d)**^b^**	0.90 (.73–1.12)	.3487	…	**…**	…	**…**
Need for ICU admission	2.13 (.79–5.76)	.1380	…	…	…	…
Need for CRRT after *P. aeruginosa* BSI**^a^**	…		…	…	…	…

The reported *P* values are derived from Cox regression analysis. Bold values indicate statistical significance at the predefined level (α = .05). The variables hospital ward, source of bloodstream infection, and susceptibility profile were excluded from the analysis. The clinical presentation variable was replaced with the binary variable septic shock (Yes vs No). The variables time to source control, need for ICU admission, need for CRRT after *P. aeruginosa* BSI, and time to appropriate antibiotic therapy were treated as time-dependent. For 3 patients with missing ICU discharge dates, the hospital discharge date was used to define the end of ICU exposure.

Abbreviations: aHR, adjusted hazard ratio; BSI, bloodstream infection; CRRT, continuous renal replacement therapy; HIV, human immunodeficiency virus; HR, hazard ratio; ICU, intensive care unit; IQR, interquartile range.

^a^Analysis not valid due to an insufficient number of events.

^b^Analysis conducted on a total of 172 patients who underwent source control during the study period.

^c^Variable treated as fixed due to its clinical relevance, a median initiation time of 0 d (IQR: 0–1), the lack of a significant effect of time to appropriate antibiotic therapy (*P* = .1143), and the absence of interaction with time (*P* = .1525) in the Cox model, ensuring methodological consistency.

^d^Analysis conducted on a total of 578 patients who received appropriate antibiotic therapy.

^e^Within 30 d before *P. aeruginosa* BSI onset.

^f^Within 90 d before *P. aeruginosa* BSI onset.

**Table 3.** Multivariable Analyses of Factors Associated With 30-d All-Cause Mortality: Time-Dependent Source Control (Model B), 4-d Landmark Analysis (Model C, n = 562), Shared Frailty Model (Model D), and Model With Source Control Regardless of Whether It Was Deemed Necessary (Model E)

	Multivariable Backward (Model B)		Landmark Analysis (Model C, n = 562)		Shared Frailty Model (Model D)		Model E	
Variable	aHR (95% CI)	*P* Value	aHR (95% CI)	*P* Value	aHR (95% CI)	*P* Value	aHR (95% CI)	*P* Value
Source control status (vs Deemed necessary but not performed early)				.**0142**		**<**.**0001**		
Deemed necessary and adequately performed early			0.35 (.14–.90)	.**0293**	0.25 (.11–.58)	.**0013**		
Deemed unnecessary			1.23 (.72–2.13)	.4519	1.56 (1.01–2.39)	.**0439**		
Source control (vs Deemed necessary but not adequately performed)**^a^**		.**0001**						
Deemed necessary and adequately performed	0.33 (.15–.73)	.**0066**						
Deemed unnecessary	1.42 (.88–2.29)	.1513						
Deemed necessary and adequately performed source control (vs Not)							0.19 (.09–.40)	**<**.**0001**
Age (y)	1.03 (1.02–1.05)	**<**.**0001**	1.03 (1.01–1.05)	.**0005**	1.03 (1.02–1.04)	**<**.**0001**	1.03 (1.02–1.04)	**<**.**0001**
Corticosteroid therapy	1.93 (1.32–2.83)	.**0008**	1.46 (.87–2.43)	.1484	2.03 (1.43–2.88)	**<**.**0001**	2.00 (1.41–2.82)	**<**.**0001**
Previous *P. aeruginosa* colonization	—	—	0.52 (.29–.91)	.**0227**	0.59 (.39–.89)	.**0116**	0.61 (.41–.92)	.**0170**
Septic Shock	3.32 (2.32–4.76)	**<**.**0001**	2.44 (1.51–3.96)	.**0003**	4.01 (2.87–5.59)	**<**.**0001**	3.83 (2.76–5.31)	**<**.**0001**
Appropriate antibiotic therapy**^b^**	0.30 (.19–.47)	**<**.**0001**	0.41 (.21–.78)	.**0067**	0.26 (.18–.39)	**<**.**0001**	0.28 (.19–.41)	**<**.**0001**

The reported *P* values are derived from Cox regression analysis. Bold values indicate statistical significance at the predefined level (α = .05). An empty cell indicates variables not included in the model, while a dash (—) indicates variables not selected by the model.

Abbreviations: aHR, adjusted hazard ratio; BSI, bloodstream infection; CRRT, continuous renal replacement therapy; HR, hazard ratio; ICU, intensive care unit.

^a^Treated as time-dependent, see Methods section.

^b^Variable treated as fixed due to its clinical relevance, a median initiation time of 0 d (IQR: 0–1), the lack of a significant effect of time to appropriate antibiotic therapy (*P* = .1143), and the absence of interaction with time (*P* = .1525) in the Cox model, ensuring methodological consistency.

### Clinical Characteristics of Patients in Whom Source Control Was Deemed Necessary and Differences Between Those Who Underwent the Procedure and Those Who Did Not

Table 4 presents the clinical characteristics of the 217 patients for whom source control was deemed necessary. No statistically significant differences were observed between those who received early adequate source control (n = 107) and those who did not (n = 110), except for a higher prevalence of intermittent dialysis in patients who did not receive early adequate source control (10.9% vs 3.7%, *P* = .0433). In the Cox multivariable regression model (Table 5, model A2) 30-day all-cause mortality was associated with septic shock (aHR 6.16; CI 3.03–12.51, *P* < .0001). Conversely, early adequate source control (aHR 0.22; CI .09–.50, *P* = .0004) and male sex (aHR 0.45, CI .22–.90, *P* = .0240) were the only factors associated with improved outcomes. Similar results were observed in models B2, C2, and D2 (Supplementary Table 3).

**Table 4.** Demographic and Clinical Characteristics of the Study Population With Source Control Deemed Necessary (n = 217); Comparison Between Patients Who Received Early Adequate Source Control and Those Who Did Not

Variables	Total n = 217	Early Adequate Source Control n = 107	No Early Adequate Source Control n = 110	*P* Value
Age (y), median (IQR)	67 (56–76)	66 (55–74)	69.5 (57–77)	.2021
Male sex, n (%)	132 (60.8)	72 (67.3)	60 (54.6)	.0545
Hospital ward at the time of *P. aeruginosa* BSI onset, n (%)	…	…	…	.4920
Internal medicine	103 (47.5)	47 (43.9)	56 (50.9)	
Surgery	27 (12.4)	12 (11.2)	15 (13.6)	
Oncology-Hematology	23 (10.6)	11 (10.3)	12 (10.9)	
ICU	63 (29.0)	36 (33.6)	27 (24.6)	
Others	1 (0.5)	1 (0.9)	0 (0.0)	
Underlying diseases, n (%)				
Cardiovascular disease	71 (32.7)	34 (31.8)	37 (33.6)	.7702
Neurological disease	55 (25.4)	31 (29.0)	24 (21.8)	.2258
Diabetes mellitus	38 (17.5)	15 (14.0)	23 (20.9)	.1818
Solid malignancy	48 (22.1)	23 (21.5)	25 (22.7)	.8270
Gastrointestinal disease	39 (18.0)	21 (19.6)	18 (16.4)	.5315
Hematological malignancy	35 (16.1)	14 (13.1)	21 (19.1)	.2291
Chronic renal failure	40 (18.4)	16 (15.0)	24 (21.8)	.1923
Chronic obstructive pulmonary disease	24 (11.1)	9 (8.4)	15 (13.6)	.2198
Hematopoietic stem cell transplant	11 (5.1)	5 (4.7)	6 (5.5)	.7930
Solid organ transplant	12 (5.5)	4 (3.7)	8 (7.3)	.2548
Liver cirrhosis	4 (1.8)	2 (1.9)	2 (1.8)	1.0000**^f^**
Splenectomy	5 (2.3)	2 (1.9)	3 (2.7)	1.0000**^f^**
HIV	3 (1.4)	2 (1.9)	1 (0.9)	.6181**^f^**
Charlson comorbidity index, median (IQR)	4 (2–6)	4 (2–6)	4 (2–6)	.3156
Corticosteroid therapy	48 (22.1)	28 (26.2)	20 (18.2)	.1564
Chemotherapy	28 (12.9)	13 (12.2)	15 (13.6)	.7439
Other immunosuppressive therapy	29 (13.4)	14 (13.1)	15 (13.6)	.9049
Neutropenia	18 (8.3)	8 (7.5)	10 (9.1)	.6664
Intermittent dialysis treatment	16 (7.4)	4 (3.7)	12 (10.9)	.**0433**
Invasive procedures^c^, n (%)				
Central venous catheter	157 (72.4)	82 (76.6)	75 (68.2)	.1639
Urinary catheter	134 (61.8)	67 (62.6)	67 (60.9)	.7958
Any surgery	49 (22.6)	23 (21.5)	26 (23.6)	.7061
Percutaneous endoscopic gastrostomy	13 (6.0)	6 (5.6)	7 (6.4)	.8145
Previous *P. aeruginosa* colonization^d^, n (%)	66 (30.4)	34 (31.8)	32 (29.1)	.6673
Source of bloodstream infection^a^, n (%)	…	…	…	.0969**^f^**
CVC-related BSI	144 (66.4)	80 (74.8)	64 (58.2)	
HAP/VAP	1 (0.5)	0 (0.0)	1 (0.9)	
Urinary tract infections	28 (12.9)	14 (13.1)	14 (12.7)	
Intra-abdominal infections	23 (10.6)	8 (7.5)	15 (13.6)	
Skin and soft tissue infection	11 (5.1)	3 (2.8)	8 (7.3)	
Osteomyelitis	3 (1.4)	1 (0.9)	2 (1.8)	
Central nervous system infection	1 (0.5)	0 (0.0)	1 (0.9)	
Others^e^	6 (2.7)	1 (0.9)	5 (4.6)	
Susceptibility profile^b^, n (%)	…	…	…	.4435**^f^**
MDR	22 (10.3)	10 (9.4)	12 (11.2)	
XDR	12 (5.6)	4 (3.8)	8 (7.5)	
PDR	1 (0.5)	0 (0.0)	1 (0.9)	
Clinical presentation, n (%)	…	…	…	.8642
Sepsis	105 (48.4)	52 (48.6)	53 (48.2)	
Septic Shock	42 (19.4)	22 (20.6)	20 (18.2)	
No sepsis	70 (32.3)	33 (30.8)	37 (33.6)	
Appropriate antibiotic therapy, n (%)	198 (91.2)	100 (93.5)	98 (89.1)	.2552
Outcomes, n (%)				
Need for ICU admission	6 (2.8)	3 (2.8)	3 (2.7)	1.0000**^f^**
Need for CRRT after *P. aeruginosa* BSI	4 (1.8)	1 (0.9)	3 (2.7)	.6216**^f^**
30-d all-cause mortality	33 (15.2)	7 (6.5)	26 (23.6)	.**0005**
Recurrence of BSI	6 (2.8)	2 (1.9)	4 (3.6)	.6833**^f^**

The reported *P* values are derived from the Wilcoxon rank sum test for continuous variables and χ^2^ or Fisher's exact test for categorical variables; bold values indicate statistical significance at the predefined level (α = .05). One patient in the early adequate source control group had missing age data, which was imputed using the median for subsequent analyses. The median and interquartile range reported in the table were identical before and after imputation, while the *P* value was virtually unchanged (reported: available cases; after imputation: *P* = .2029). No other missing values were present in this table.

Abbreviations: BSI, bloodstream infection; CRRT, continuous renal replacement therapy; CVC, central venous catheter; ICU, intensive care unit; IQR, interquartile range; HAP, hospital-acquired pneumonia; HIV, human immunodeficiency virus; MDR, multidrug resistant; PDR, pandrug-resistant; VAP, ventilator-associated pneumonia; XDR, extensively drug resistant.

^a^Test calculated using Monte Carlo simulation.

^b^Analysis conducted on a total of 213 patients with a known susceptibility profile.

^c^Within 30 d before *P. aeruginosa* BSI onset.

^d^Within 90 d before *P. aeruginosa* BSI onset.

^e^Includes mediastinitis, endocarditis, infection of spinal fixation hardware, community-acquired pneumonia, infection of prosthetic femur, lung abscess, pleural empyema, LVAD infection.

^f^Fisher's exact test.

**Table 5.** Univariable, Multivariable, and Backward Selection Multivariable Analyses of Factors Associated With 30-d All-Cause Mortality in the Study Population With Source Control Deemed Necessary (n = 217)

	Univariable		Multivariable		Multivariable Backward (Model A2)	
Variable	HR (95% CI)	*P* Value	aHR (95% CI)	*P* Value	aHR (95% CI)	*P* Value
Age (y)	1.02 (1.00–1.05)	.0704	1.02 (.99–1.04)	.1632	…	**…**
Male sex	0.45 (.22–.89)	.**0224**	0.45 (.22–.90)	.**0245**	0.45 (.22–.90)	.**0240**
Cardiovascular disease	1.72 (.87–3.41)	.1209	…	**…**	…	**…**
Neurological disease	1.45 (.70–2.99)	.3145	…	**…**	…	**…**
Diabetes mellitus	0.74 (.29–1.93)	.5424	…	**…**	…	**…**
Solid malignancy	0.92 (.40–2.13)	.8517	…	**…**	…	**…**
Gastrointestinal disease	0.65 (.23–1.84)	.4112	…	**…**	…	**…**
Hematological malignancy	0.36 (.09–1.49)	.1564	…	**…**	…	**…**
Chronic renal failure	0.99 (.41–2.39)	.9729	…	**…**	…	**…**
Chronic obstructive pulmonary disease	0.87 (.26–2.84)	.8121	…	**…**	…	**…**
Hematopoietic stem cell transplant**^a^**	…	…	…	**…**	…	**…**
Solid organ transplant**^a^**	…	…	…	**…**	…	**…**
Liver cirrhosis**^a^**	…	…	…	**…**	…	**…**
Splenectomy**^a^**	…	…	…	**…**	…	**…**
HIV**^a^**	…	…	…	**…**	…	**…**
Charlson comorbidity index	1.06 (.95–1.18)	.3231	…	**…**	…	**…**
Corticosteroid therapy	0.72 (.30–1.75)	.4693	…	**…**	…	**…**
Chemotherapy	0.70 (.21–2.29)	.5540	…	**…**	…	**…**
Other immunosuppressive therapy**^a^**	…	…	…	…	…	…
Neutropenia	0.81 (.19–3.38)	.7696	…	…	…	**…**
Intermittent dialysis**^a^**	…	**…**	…	**…**	…	**…**
Central venous catheter^e^	0.59 (.29–1.18)	.1371	…	**…**	…	**…**
Urinary catheter^e^	1.85 (.83–4.10)	.1309	…	…	…	**…**
Any surgery^e^	1.43 (.68–3.00)	.3509	…	…	…	**…**
Percutaneous endoscopic gastrostomy^e^	1.53 (.47–5.03)	.4823	…	…	…	…
Previous *P. aeruginosa* colonization^f^	0.63 (.28–1.40)	.2582	…	…	…	**…**
Septic shock	4.54 (2.29–9.00)	**<**.**0001**	6.16 (3.02–12.57)	**<**.**0001**	6.16 (3.03–12.51)	**<**.**0001**
Deemed necessary and adequately performed early source control	0.26 (.11–.60)	.**0017**	0.21 (.09–.50)	.**0004**	0.22 (.09–.50)	.**0004**
Time to source control (d)**^b^**	0.90 (.73–1.12)	.3487	…	…	…	…
Appropriate antibiotic therapy^c^	0.51 (.20–1.32)	.1663	…	…	…	…
Time to appropriate antibiotic therapy (d)**^d^**	0.79 (.56–1.11)	.1727	…	…	…	…
Need for ICU admission**^a^**	…	**…**	…	…	…	…
Need for CRRT after *P. aeruginosa* BSI**^a^**	…	…	…	…	…	…

The reported *P* values are from the Cox regression analysis. Bold values are significant at the selected level of significance (α = .05). Variables source of bloodstream infection, hospital ward at the time of *P. aeruginosa* BSI onset, susceptibility profile, and recurrence of BSI were excluded from the analysis. Variables time to source control and time to appropriate antibiotic therapy were treated as time-dependent.

Abbreviations: aHR, adjusted hazard ratio; BSI, bloodstream infection; CRRT, continuous renal replacement therapy; HR, hazard ratio; ICU, intensive care unit.

^a^Analysis not done due to insufficient events.

^b^Analysis conducted on a total of 172 patients who underwent source control during the study period.

^c^ Appropriate antibiotic therapy was treated as fixed covariate due to its clinical relevance, the median initiation time of 0 (IQR: 0–2) day, the lack of a significant effect of time to appropriate antibiotic therapy (*P* = .1727), and the absence of interaction with time (*P* = .3189) in the Cox model.

^d^Analysis conducted on a total of 198 patients who received appropriate antibiotic therapy.

^e^Within 30 d before *P. aeruginosa* BSI onset.

^f^Within 90 d before *P. aeruginosa* BSI onset.

We also performed a PS-based analysis, in which 70 pairs of patients who received early adequate source control were matched to those who did not. The standardized differences between the 2 groups were ≤0.2 for all variables except hematological malignancy, indicating that the groups were well balanced. The Cox proportional hazards model in the PS-matched cohort, adjusted for hematological malignancy, confirmed that early adequate source control was associated with a lower 30-day all-cause mortality rate (aHR 0.15, 95% CI .04–.51, *P* = .0024) compared to patients who did not receive it. The results in the 4-day landmark analysis were largely consistent with the PS findings.

### Impact of Source Control and Antibiotic Therapy on Mortality

Among patients with source control deemed necessary who survived at least 4 days from BSI onset, 30-day mortality rates were low in those undergoing early adequate source control, irrespective of antibiotic appropriateness (0% vs 6.2%, *P* = 1.0000). In contrast, among patients who did not receive early adequate source control, mortality was 16.5% among those who received appropriate antibiotic therapy and 22.2% in those who did not; this difference was not statistically significant (*P* = .6475) (Supplementary Figure 1).

## DISCUSSION

The key findings of this multicenter cohort study can be summarized as follows: (1) approximately one in 3 patients with *P. aeruginosa* BSI required source control; (2) early adequate source control was associated with a significantly lower risk of 30-day all-cause mortality; and (3) in the exploratory analysis restricted to patients requiring source control, mortality was numerically lower with appropriate antibiotic therapy among patients without early adequate source control, whereas mortality remained low overall among those who received early adequate source control.

*P. aeruginosa* infections are associated with high mortality, as highlighted in a large observational study on sepsis conducted in 198 European ICUs, where it was the only pathogen independently linked to mortality [27]. Several studies confirm the poor prognosis of *P. aeruginosa* BSI, with attributable mortality rates reaching up to 60% [13–16]. In recent years, efforts to improve outcomes have primarily focused on early diagnosis [15], optimized antibiotic dosing [28], and combination therapy in both empirical [16] and targeted regimens [29]. In contrast, adequate source control-a cornerstone of infection management, encapsulated in the ancient principle *ubi pus, ibi evacua-*has received little attention [1]. Few studies have evaluated its impact [19, 20], and current guidelines make only a brief mention of its role in *P. aeruginosa* BSI [30, 31].

Yet, adequate source control remains essential for a substantial proportion of patients with *P. aeruginosa* BSI [19, 20]. A recent study by Papadimitriou-Olivgeris et al [19] reported that 50% of *P. aeruginosa* BSI cases required source control. However, their analysis considered catheter removal in all patients with a CVC, regardless of whether it was identified as the actual infection source [19]. To better reflect clinical practice and guideline-based infection management, we considered CVC removal necessary only when the catheter was confirmed as the likely infection source [2]. Using this more stringent definition, our findings indicate that approximately one-third of *P. aeruginosa* BSI cases required source control, underscoring the need for a more structured and evidence-based approach to its management.

Although the absence of early source control has been identified as an independent predictor of mortality in BSI caused by various pathogens [5–8], this critical aspect of *P. aeruginosa* BSI management has often been overlooked in mortality analyses [32–34]. To our knowledge, only one retrospective study, conducted at a single center in Switzerland and including 278 patients with *P. aeruginosa* BSI, has thoroughly addressed this issue [19], demonstrating a significant survival benefit associated with early adequate source control. In that study, 14-day mortality was markedly lower in patients who underwent source control compared to those who did not (7% vs 21%) [19]. Our study reinforces and expands these findings by providing a national multicenter perspective and including a higher proportion of patients with septic shock due to *P. aeruginosa* (20% vs 0%) [19]. Additionally, our cohort had a higher prevalence of MDR, XDR or PDR strains (overall 16.6% vs 7.0%) [19], further emphasizing the broader clinical relevance of early source control in improving patient outcomes.

Of importance, among patients who achieved early adequate source control, 30-day mortality rates were very low, with no statistically significant difference by antibiotic appropriateness (*P* = 1.0000; Supplementary  Figure 1). Conversely, among patients without early adequate source control, mortality was numerically lower in those receiving appropriate antibiotic therapy (16.5% vs 22.2%), although the difference was not statistically significant (*P* = .6475). These exploratory findings highlight that, among patients without early adequate source control, mortality remained substantial even when appropriate antibiotic therapy was administered. From a clinical perspective, these findings emphasize the importance of promptly identifying and managing the infectious focus, as recommended for other severe infections (eg, *S. aureus* and *Candida* BSI) [35–37]. In this context, infectious disease specialists play a key role, as previous studies have shown their contribution to improving infection source identification and, consequently, patient outcomes [20, 38].

Our findings regarding other mortality risk factors align with recent studies identifying older age [32, 34, 39], corticosteroid therapy [32, 34], and septic shock at presentation [32, 34, 39] as key predictors of 30-day all-cause mortality in *P. aeruginosa* BSI. Notably, in contrast with prior studies [40, 41], we observed a protective effect of prior *P. aeruginosa* colonization. This may be explained by clinicians' heightened suspicion of *P. aeruginosa* infection in colonized patients, leading to earlier interventions, including source control measures and prompt initiation of appropriate antibiotic therapy based on prior microbiological data. However, susceptibility data from previous colonizing isolates and their concordance with empirical therapy were not available; therefore, this hypothesis could not be directly assessed.

Our study has other limitations that should be acknowledged. First, multiple confounding factors may have influenced the observed association between early interventions and improved survival, including decisions to limit or withdraw care and the availability and willingness of surgeons or interventional radiologists to perform adequate source control procedures. However, our findings were confirmed in the landmark analysis, which included only patients who were alive at day 4 after *P. aeruginosa* BSI onset. Second, infection source attribution and the need for source control were assessed by the local investigators at each participating center based on the available clinical information. As a result, some patients classified as having BSI of unknown source may have had an occult catheter-related, urinary, pulmonary, gastrointestinal, or another potential focus that was not identified. The high mortality rate observed in this group comparable to that among patients for whom source control was indicated but not performed supports this hypothesis. This potential misclassification should be considered when interpreting the outcomes of patients in whom source control is deemed unnecessary and further reinforces the importance of a systematic approach to *P. aeruginosa* BSI and the need for actively identifying and addressing the infection source. Third, although PS-matching does not fully adjust for time-dependent bias, it was included just as a complementary, exploratory analysis to support the robustness of the main findings. Fourth, the use of a 4-day landmark analysis, applied to assess the interplay between source control adequacy and appropriate antibiotic therapy, helped mitigate immortal time bias by including only patients who were alive at day 4 after BSI onset. However, this approach inherently excludes early deaths and may therefore underestimate the potential benefit of early interventions in the most critically ill patients. Fifth, we collected information only on the first source control intervention. Data on additional source control procedures were not recorded; therefore, their potential impact on patient outcomes could not be assessed. Lastly, we did not assess the impact of adequate source control on the duration of appropriate antibiotic therapy [42] or its potential role in reducing the risk of in vivo antimicrobial resistance development [43]-both of which may represent additional benefits of effective source control. However, our study was not designed to address these aspects, and further research is needed to explore them.

By addressing the knowledge gap regarding the role of appropriate source control in *P. aeruginosa* BSI outcomes, our study provides valuable insights into the multifaceted approach required for its effective management. Our findings underscore the critical importance of early identification and appropriate implementation of source control in patients with *P. aeruginosa* BSI, highlighting its potential to improve patient care and reduce mortality. Further research is warranted to refine source control strategies and assess their impact on antimicrobial resistance and treatment duration.

## Supplementary Material

ofag488_Supplementary_Data

## Supplementary Data

Supplementary materials are available at *Open Forum Infectious Diseases* online. Consisting of data provided by the authors to benefit the reader, the posted materials are not copyedited and are the sole responsibility of the authors, so questions or comments should be addressed to the corresponding author.
