# Supplementary material for: Source Control in Pseudomonas aeruginosa Bloodstream Infections: An Overlooked Pillar in Reducing 30-Day All-Cause Mortality
Source: Open Forum Infect Dis. 2026 Sep 24;13(9):ofag488. doi: 10.1093/ofid/ofag488 (PMC13601804; doi:10.1093/ofid/ofag488)
Supplement: Supplementary file 1 [file ofag488_supplementary_data.zip › 7.CLEAN VERSION.docx]

**CLEAN VERSION**

**OFID-D-26-00562**

**SUPPLEMENTARY MATERIALS**

**Table S1.** Baseline clinical characteristics of patients with BSI of unknown source (n = 203)

| **Variable** | **Total**  **n=203** |
| --- | --- |
|  |  |
| **Age (years), median (IQR)** | 69 (59-80) |
| **Male sex, n (%)** | 133 (65.5) |
| **Hospital ward stay at the time of P. aeruginosa BSI onset, n (%)** |  |
| Internal medicine | 101 (49.8) |
| Surgery | 29 (14.3) |
| Oncology-Haematology | 31 (15.3) |
| ICU | 39 (19.2) |
| Others | 3 (1.5) |
| **Underlying diseases**, **n (%)** |  |
| Cardiovascular disease | 74 (36.5) |
| Neurological disease | 67 (33.0) |
| Diabetes mellitus | 37 (18.2) |
| Solid malignancy | 50 (24.6) |
| Gastrointestinal disease | 47 (23.2) |
| Haematological malignancy | 41 (20.2) |
| Chronic renal failure | 29 (14.3) |
| Chronic obstructive pulmonary disease | 21 (10.3) |
| Hematopoietic stem cell transplant | 16 (7.9) |
| Solid organ transplant | 4 (2.0) |
| Liver cirrhosis | 6 (3.0) |
| Splenectomy | 0 (0.0) |
| HIV | 3 (1.5) |
| **Charlson comorbidity index, median (IQR)** | 3 (2-5) |
| Corticosteroid therapy | 48 (23.7) |
| Chemotherapy | 34 (16.8) |
| Other immunosuppressive therapy | 21 (10.3) |
| Neutropenia | 36 (17.7) |
| Intermittent dialysis treatment | 10 (4.9) |
| **Invasive procedures^a^ ,n (%)** |  |
| Central venous catheter | 117 (57.6) |
| Urinary catheter | 140 (69.0) |
| Any surgery | 51 (25.1) |
| Percutaneous endoscopic gastrostomy | 11 (5.4) |
| **Previous *P. aeruginosa* colonization^b^, n (%)** | 27 (13.3) |
| **Susceptibility profile**, n (%)** |  |
| MDR | 9 (4.5) |
| XDR | 9 (4.5) |
| PDR | 1 (0.5) |
| **Clinical presentation at BSI onset, n (%)** |  |
| Sepsis | 88 (43.4) |
| Septic Shock | 35 (17.2) |

nsive Care Unit; **MDR**: multidrug resistant; **XDR**: extremely drug resistant.

^a^ Within 30 days before P. aeruginosa BSI onset.

^b^ Within 90 days before P. aeruginosa BSI onset.

** Analysis conducted on a total of 202 patients with a known Susceptibility profile.

**Table S2.** Demographic and clinical characteristics of the entire population with *P. aeruginosa* BSI (N=639), with a comparison based on source control status.

| **Variable** | **Total**  **N=639** | **Deemed unnecessary source control**  **n=422** | **Deemed necessary source control** | | **p-value** |
| --- | --- | --- | --- | --- | --- |
|  |  |  | **Deemed necessary and adequately performed early**  **n=107** | **Deemed necessary but not performed early**  **n=110** |  |
| **Age (years), median (IQR)** | 68 (57-78) | 69 (57-78) | 66 (55-74) | 69.5 (57-77) | 0.1677 |
| **Male sex, n (%)** | 431 (67.5) | 299 (70.9) | 72 (67.3) | 60 (54.6) | **0.0051** |
| **Hospital ward stay at the time of**  ***P. aeruginosa* BSI onset, n (%)** |  |  |  |  | 0.5318 |
| Internal medicine | 279 (43.7) | 176 (41.7) | 47 (43.9) | 56 (50.9) |  |
| Surgery | 100 (15.7) | 73 (17.3) | 12 (11.2) | 15 (13.6) |  |
| Oncology-Haematology | 71 (11.1) | 48 (11.4) | 11 (10.3) | 12 (10.9) |  |
| ICU | 183 (28.6) | 120 (28.4) | 36 (33.6) | 27 (24.6) |  |
| Others | 6 (0.9) | 5 (1.2) | 1 (0.9) | 0 (0.0) |  |
| **Underlying diseases**, **n (%)** |  |  |  |  |  |
| Cardiovascular disease | 207 (32.4) | 136 (32.2) | 34 (31.8) | 37 (33.6) | 0.9505 |
| Neurological disease | 179 (28.0) | 124 (29.4) | 31 (29.0) | 24 (21.8) | 0.2815 |
| Diabetes mellitus | 125 (19.6) | 87 (20.6) | 15 (14.0) | 23 (20.9) | 0.2844 |
| Solid malignancy | 113 (20.8) | 85 (20.1) | 23 (21.5) | 25 (22.7) | 0.8228 |
| Gastrointestinal disease | 110 (17.2) | 71 (16.8) | 21 (19.6) | 18 (16.4) | 0.7643 |
| Haematological malignancy | 104 (16.3) | 69 (16.4) | 14 (13.1) | 21 (19.1) | 0.4864 |
| Chronic renal failure | 99 (15.5) | 59 (14.0) | 16 (15.0) | 24 (21.8) | 0.1273 |
| Chronic obstructive pulmonary disease | 74 (11.6) | 50 (11.9) | 9 (8.4) | 15 (13.6) | 0.4646 |
| Hematopoietic stem cell transplant | 35 (5.5) | 24 (5.7) | 5 (4.7) | 6 (5.5) | 0.9186 |
| Solid organ transplant | 28 (4.4) | 16 (3.8) | 4 (3.7) | 8 (7.3) | 0.2741^F^ |
| Liver cirrhosis | 21 (3.3) | 17 (4.0) | 2 (1.9) | 2 (1.8) | 0.4494^F^ |
| Splenectomy | 10 (1.6) | 5 (1.2) | 2 (1.9) | 3 (2.7) | 0.4575^F^ |
| HIV | 8 (1.3) | 5 (1.2) | 2 (1.9) | 1 (0.9) | 0.7591^F^ |
| **Charlson comorbidity index, median (IQR)** | 4 (2-6) | 3 (2-6) | 4 (2-6) | 4 (2-6) | 0.3623 |
| Corticosteroid therapy | 142 (22.2) | 94 (22.3) | 28 (26.2) | 20 (18.2) | 0.3672 |
| Chemotherapy | 82 (12.8) | 54 (12.8) | 13 (12.2) | 15 (13.6) | 0.9471 |
| Other immunosuppressive therapy | 72 (11.3) | 43 (10.2) | 14 (13.1) | 15 (13.6) | 0.4817 |
| Neutropenia | 70 (11.0) | 52 (12.3) | 8 (7.5) | 10 (9.1) | 0.2826 |
| Intermittent dialysis treatment | 36 (5.6) | 20 (4.7) | 4 (3.7) | 12 (10.9) | **0.0285** |
| **Invasive procedures^a^,n (%)** |  |  |  |  |  |
| Central venous catheter | 411 (64.3) | 254 (60.2) | 82 (76.6) | 75 (68.2) | **0.0042** |
| Urinary catheter | 419 (65.6) | 285 (67.5) | 67 (62.6) | 67 (60.9) | 0.3339 |
| Any surgery | 172 (26.9) | 123 (29.2) | 23 (21.5) | 26 (23.6) | 0.1952 |
| Percutaneous endoscopic gastrostomy | 40 (6.3) | 27 (6.4) | 6 (5.6) | 7 (6.4) | 0.9544 |
| ~~CRRT~~ | ~~33 (5.2)~~ | ~~25 (5.9)~~ | ~~4 (3.7)~~ | ~~4 (3.6)~~ | ~~0.4804~~ |
| **Previous *P. aeruginosa* colonization^b^, n (%)** | 171 (26.8) | 105 (24.9) | 34 (31.8) | 32 (29.1) | 0.2955 |
| **Source of bloodstream infection*, n (%)** |  |  |  |  | **<0.0001**^F^ |
| Unknown source | 203 (31.8) | 203 (48.1) | 0 (0.0) | 0 (0.0) |  |
| CVC-related BSI | 144 (22.5) | 0 (0.0) | 80 (74.8) | 64 (58.2) |  |
| HAP**/**VAP | 130 (20.3) | 129 (30.5) | 0 (0.0) | 1 (0.9) |  |
| Urinary tract infections | 73 (11.4) | 45 (10.7) | 14 (13.1) | 14 (12.7) |  |
| Intra-abdominal infections | 44 (6.9) | 21 (5.0) | 8 (7.5) | 15 (13.6) |  |
| Skin and soft tissue infection | 26 (4.1) | 15 (3.6) | 3 (2.8) | 8 (7.3) |  |
| Osteomyelitis | 3 (0.5) | 0 (0.0) | 1 (0.9) | 2 (1.8) |  |
| Central nervous system infection | 2 (0.3) | 1 (0.2) | 0 (0.0) | 1 (0.9) |  |
| Others^c^ | 14 (2.2) | 8 (1.9) | 1 (0.9) | 5 (4.6) |  |
| **Susceptibility profile**, n (%)** |  |  |  |  | 0.7276^F^ |
| MDR | 57 (9.1) | 35 (8.4) | 10 (9.4) | 12 (11.2) |  |
| XDR | 40 (6.4) | 28 (6.7) | 4 (3.8) | 8 (7.5) |  |
| PDR | 7 (1.1) | 6 (1.4) | 0 (0.0) | 1 (0.9) |  |
| **Clinical presentation at BSI onset, n (%)** |  |  |  |  | 0.9644 |
| Sepsis | 312 (48.8) | 207 (49.1) | 52 (48.6) | 53 (48.2) |  |
| Septic Shock | 129 (20.2) | 87 (20.6) | 22 (20.6) | 20 (18.2) |  |
| **Appropriate antibiotic therapy, n (%)** | 578 (90.5) | 380 (90.1) | 100 (93.5) | 98 (89.1) | 0.4878 |
| **Outcomes, n (%)** |  |  |  |  |  |
| Need for ICU admission | 23 (3.6) | 17 (4.0) | 3 (2.8) | 3 (2.7) | 0.8572^F^ |
| Need for CRRT after *P.aeruginosa* BSI | 20 (3.1) | 16 (3.8) | 1 (0.9) | 3 (2.7) | 0.3874^F^ |
| 30-day all-cause mortality | 153 (23.9) | 120 (28.4) | 7 (6.5) | 26 (23.6) | **<0.0001** |
| **Time to death or discharge (days), median (IQR)** | 17 (9-35) | 16 (8-33) | 22 (12-41) | 20 (11-36) | **0.0032** |

The reported p-values for three-category comparisons are derived from the Wilcoxon Rank Sum test for continuous variables and the Chi-Square or Fisher’s Exact test for categorical variables. Bold values indicate statistical significance at the predefined level (α = 0.05). One patient with adequately performed early

source control had missing age data, which was imputed using the median for subsequent analyses. The median and interquartile range reported in the table were identical before and after imputation, while the p-value was virtually unchanged (reported: available cases; after imputation: p = 0.1689). No other missing values were present in this table.

**ABBREVIATIONS:** **BSI:** bloodstream infection; **CRRT:** Continuous renal replacement therapy; **ICU**: Intensive Care Unit; **MDR:** multidrug resistant; **XDR:** extremely drug resistant.

* Test calculated using Monte Carlo simulation.
** Analysis conducted on a total of 629 patients with a known Susceptibility profile.
^a^ Within 30 days before P. aeruginosa BSI onset.

^b^ Within 90 days before P. aeruginosa BSI onset.

^c^ Includes Mediastinitis, Endocarditis, Vertebral fixations infection, Community-acquired pneumonia, Infection of prosthetic femur, Lung abscess, Pleural empyema, LVAD infection.

^F^ Fisher’s Exact test.

**Table S3**. Multivariable analyses of factors associated with 30-day all-cause mortality in the study population with source control deemed necessary: time-dependent source control (Model B2), 4-day landmark analysis (Model C2, n=204), and shared frailty model (Model D2).

|  | **Multivariable backward (Model B2)** | | **Landmark analysis (Model C2, n=204)** | | **Shared frailty model (Model D2)** | |
| --- | --- | --- | --- | --- | --- | --- |
| **Variable** | **aHR (95% CI)** | **p-value** | **aHR (95% CI)** | **p-value** | **aHR (95% CI)** | **p-value** |
| Early adequate source control |  |  | 0.32 (0.12-0.83) | **0.0198** | 0.21 (0.09-0.49) | **0.0004** |
| Adequate source control* | 0.20 (0.09-0.46) | **0.0001** |  |  |  |  |
| Male sex | - | - | 0.41 (0.17-0.95) | **0.0377** | 0.43 (0.20-0.92) | **0.0302** |
| Septic Shock | 3.73 (1.81-7.69) | **0.0004** | 3.52 (1.44-8.61) | **0.0057** | 7.13 (3.39-15.01) | **<0.0001** |

The reported p-values are from the Cox regression analysis. Bold values are significant at the selected level of significance (α = 0.05). Empty cell indicates variables not included in model, dash indicates variables not selected by the model.

**ABBREVIATIONS:** **aHR:** Adjusted Hazard Ratio; **CI:** Confidence Interval

* Treated as time dependent.

**Figure 1s.** 30-day all-cause mortality among patients surviving ≥4 days, stratified by early adequate source control and appropriate antibiotic therapy.
